# Supplementary material for: Impact of pathogen genetics on clinical phenotypes in a population of Talaromyces marneffei from Vietnam
Source: bioRxiv. 2023 Apr 1:2023.03.30.534926. Preprint. [Version 1] doi: 10.1101/2023.03.30.534926 (PMC10081260; doi:10.1101/2023.03.30.534926)
Supplement: 1 [file NIHPP2023.03.30.534926v1-supplement-1.pdf]

bioRxiv preprint doi: https://doi.org/10.1101/2023.03.30.534926; this version posted April 1, 2023. The copyright holder for this preprint (which was not certified by peer review) is the author/funder, who has granted bioRxiv a license to display the preprint in perpetuity. It is made available under aCC-BY 4.0 International license.

664 Supplemental Table 1. Functional enrichment of regions under selection across clades.

Category	Test	GO Term	Adjusted p-value
Sub-telomeric	CLR	GO:0015980	0.04
Sub-telomeric	CLR	GO:0042357	0.04
Non sub-telomeric	CLR	GO:0015980	0.006
Non sub-telomeric	CLR	GO:0045333	0.006
Non sub-telomeric	CLR	GO:0015711	0.02
Non sub-telomeric	CLR	GO:0006811	0.03
Non sub-telomeric	CLR	GO:0010468	0.03
Non sub-telomeric	Dxy	GO:0009061	0.05

bioRxiv preprint doi: https://doi.org/10.1101/2023.03.30.534926; this version posted April 1, 2023. The copyright holder for this preprint (which was not certified by peer review) is the author/funder, who has granted bioRxiv a license to display the preprint in perpetuity. It is made available under aCC-BY 4.0 International license.

Supplemental Figure 1. Recombination rates per chromosome.

Recombination rates (p/bp) as calculated by Ldhelmet, for a subset of 50 isolates.

Supplemental Figure 2. Population structure and geographical clades.

A) Population structure as determined by splitstree identifies two distinct clades, grouped by geography. B) Maximum likelihood phylogeny of patient isolates, with minimum inhibitory concentrations of itraconazole (ITR, blue) and amphotericin B (AMB, orange), visualized around the outer perimeter of the phylogeny.

Supplemental Figure 3. Linkage disequilibrium decay.

bioRxiv preprint doi: https://doi.org/10.1101/2023.03.30.534926; this version posted April 1, 2023. The copyright holder for this preprint (which was not certified by peer review) is the author/funder, who has granted bioRxiv a license to display the preprint in perpetuity. It is made available under aCC-BY 4.0 International license.

Linkage disequilibrium decay over 250 kb for northern (orange) and southern (blue) clades, as well as all isolates combined (grey).

Supplemental Figure 4. Initial fungal burden and clearance rate by clade.

A) Rate of clearance (Slope) by the infecting isolate clade. Displayed as −1(gradient). B) Blood Log_{10} CFU/mL (fungal burden) by infecting isolate clade.

Supplemental Figure 5. Minimum inhibitory concentrations of itraconazole and amphotericin B.

A) Histogram displaying the minimum inhibitory concentrations of itraconazole (ITR). B) Histogram displaying the minimum inhibitory concentrations of amphotericin B (AMB).
